# Sequential Autologous Stem Cell Transplantation and Ciltacabtagene Autoleucel for Concurrent Multiple Myeloma and Presumptive Immunoglobulin Heavy- and Light-Chain Amyloidosis in a Patient With Systemic Lupus Erythematosus

**DOI:** 10.7759/cureus.115042

**Published:** 2026-08-23

**Authors:** Riddhi Patel, Yash Patel, Harshitha Tallapally, Chetan Shah

**Affiliations:** 1 Medicine, Gujarat Cancer Society (GCS) Medical College, Hospital, and Research Centre, Ahmedabad, IND; 2 Medicine, Smt. Nathiba Hargovandas Lakhmichand (NHL) Municipal Medical College, Ahmedabad, IND; 3 Medicine, Osmania Medical College, Hyderabad, IND; 4 Nephrology, Specialty MedConsultants (SMC) Physicians, Ventnor, USA

**Keywords:** ahl amyloidosis, autologous stem cell transplantation, car t-cell therapy, ciltacabtagene autoleucel, multiple myeloma, nephrotic syndrome, plasma cell dyscrasia, systemic lupus erythematosus

## Abstract

The co-occurrence of multiple myeloma (MM), systemic immunoglobulin light chain (AL) and heavy-and-light-chain (AHL) amyloidosis, and systemic lupus erythematosus (SLE) presents a highly complex therapeutic challenge. While B-cell maturation antigen (BCMA)-directed chimeric antigen receptor (CAR) T-cell therapies have reshaped the treatment of MM, their safety and efficacy in patients with underlying autoimmune diatheses and severe organ-damaging amyloidosis remain sparsely documented. We report the case of a 67-year-old African American male with a history of SLE who presented with declining renal function and nephrotic-range proteinuria. A renal biopsy in month one revealed AL amyloidosis (Lambda type) with heavy chain (IgG1) co-deposition suggestive of AHL amyloidosis. A subsequent bone marrow biopsy confirmed concurrent MM. The patient was treated with induction chemotherapy (carfilzomib, daratumumab, lenalidomide), followed by an autologous stem cell transplant (ASCT) in month eight. He subsequently received ciltacabtagene autoleucel (cilta-cel) in month 14. By month 16, a bone marrow biopsy confirmed complete marrow remission. At his month 22 follow-up, the patient remained off all antineoplastic agents, demonstrating substantial organ response with near-complete resolution of proteinuria (9.4 mg/dL) and stabilized chronic kidney disease (CKD) stage 3b. Persistent, mild cytopenias and an elevated B-type natriuretic peptide (BNP) prompted ongoing post-CAR-T supportive care and evaluation for cardiac amyloidosis. This case highlights the feasibility of combining ASCT with BCMA-directed CAR T-cell therapy and its association with continued hematologic and renal improvement in highly complex patients with overlapping plasma cell dyscrasias, amyloid deposition, and autoimmune disease.

## Introduction

Systemic amyloidosis is a rare disease in which misfolded fibrillar proteins accumulate as amyloid deposits in multiple organs, interfering with normal function. The most common subtype is immunoglobulin light chain (AL) amyloidosis, which is driven by an underlying plasma cell dyscrasia such as multiple myeloma (MM) [1]. Rarely, both heavy and light chains co-deposit, a condition known as heavy-and-light-chain (AHL) amyloidosis [2,3]. This condition often rapidly progresses to end-stage renal disease (ESRD) due to massive proteinuria and nephrotoxicity, so traditionally had a poor renal prognosis.

The management of such patients is intractable when accompanied by underlying autoimmune conditions like systemic lupus erythematosus (SLE), which can independently cause renal damage (lupus nephritis) and limit tolerance to immunomodulatory therapies [4,5].

Recently, ciltacabtagene autoleucel (cilta-cel), a B-cell maturation antigen (BCMA)-directed chimeric antigen receptor (CAR) T-cell therapy, has demonstrated unprecedented efficacy in treating relapsed/refractory MM [6,7]. However, real-world data detailing its utilization in patients with combined MM, SLE, and tissue-damaging amyloidosis is exceedingly rare, representing a significant clinical knowledge gap. We present the clinical course of a patient who attained marrow remission and noteworthy renal recovery following sequential autologous stem cell transplant (ASCT) and cilta-cel therapy, distinguishing the challenges of navigating confirmed AL and presumptive AHL amyloidosis in this overlap setting.

## Case presentation

Patient characteristics and initial diagnosis

A 67-year-old African American (as identified via electronic health records) male with a history of hypertension, anti-nuclear antibody (ANA)-positive SLE, arthritis, and sciatica was assessed for massive proteinuria and deteriorating renal function. Due to limitations in retrospective medical record access, granular SLE serologies (e.g., anti-dsDNA, C3/C4 levels) and formal disease activity scores at the time of presentation were unavailable.

**Figure 1:**
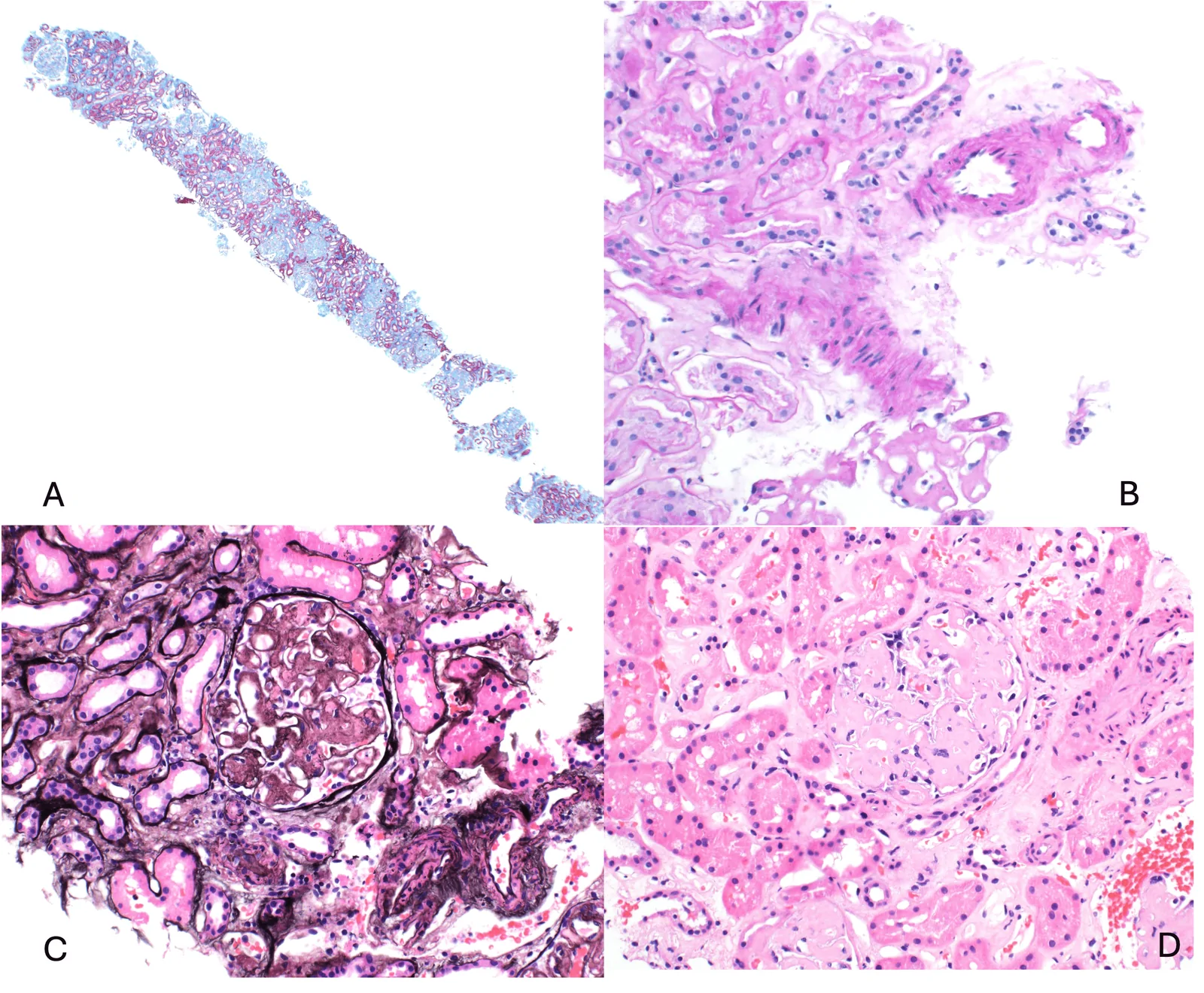
Light microscopy of renal amyloidosis Composite image demonstrating (A) low power view of the renal cortex, (B) a normal artery, and (C, D) pale amorphous material expanding the mesangium and capillary loops. Original magnification x400.

In month one, a percutaneous renal biopsy was performed, demonstrating a low-power view of the renal cortex (Figure 1A), a normal artery (Figure 1B), and pale amorphous material expanding the mesangium and capillary loops (Figures 1C, 1D).

**Figure 2:**
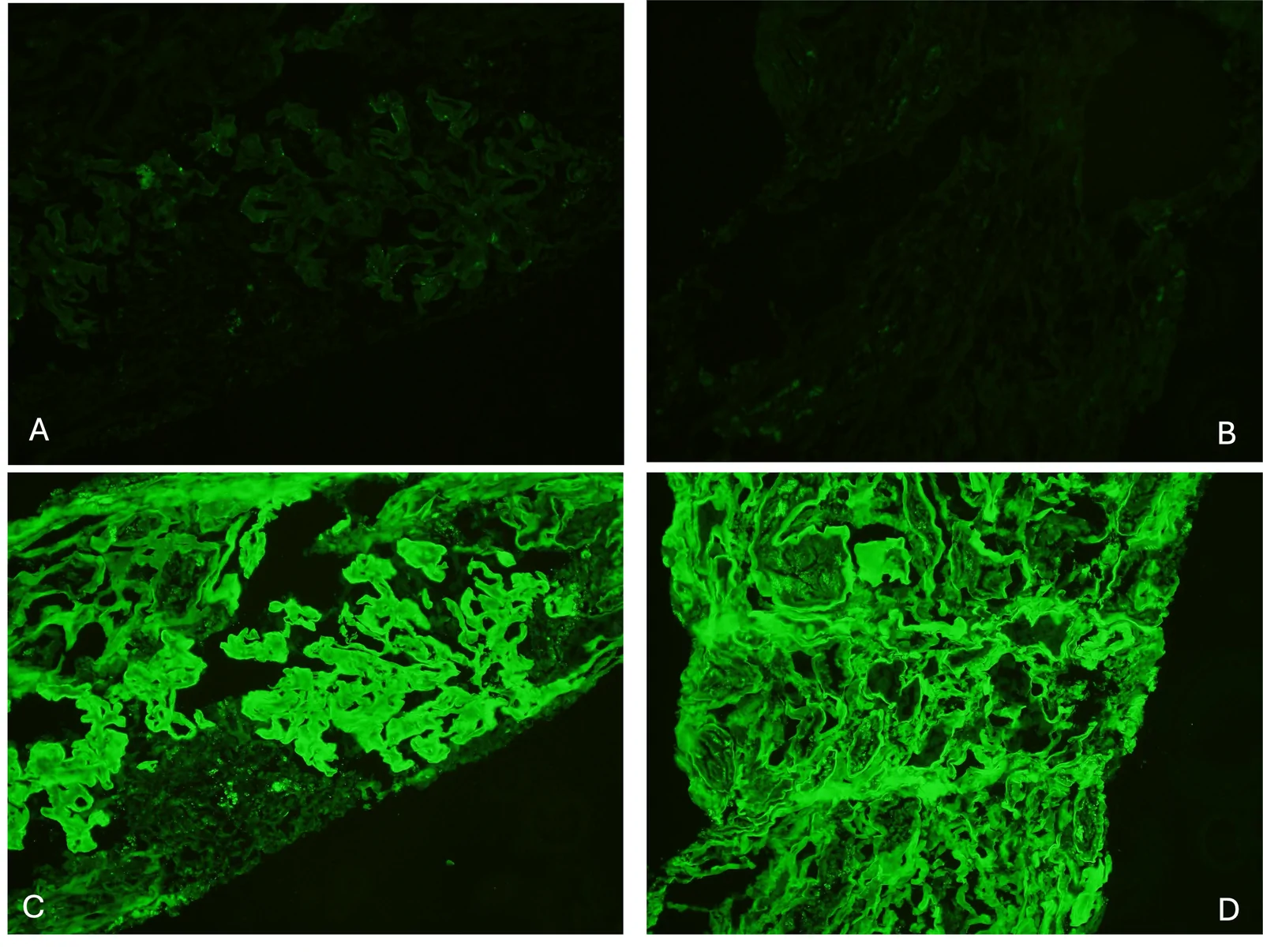
Immunofluorescence demonstrating lambda restriction Direct immunofluorescence of the glomerulus demonstrating (A, B) negative staining for kappa light chains and (C, D) concurrent strong granular positivity for lambda light chains within the mesangial and capillary loop amyloid deposits. Original magnification x400.

Direct immunofluorescence revealed negative kappa staining (Figure 2A, 2B), and concurrent strong granular positivity for lambda light chains (Figure 2C, 2D) alongside IgG1 heavy chain staining (up to 2+) within the amyloid deposits, raising the possibility of a heavy chain component (i.e., AHL amyloidosis). Importantly, the biopsy did not demonstrate a "full-house" immunofluorescence pattern, proliferative lesions, or tubuloreticular inclusions, supporting amyloidosis rather than concomitant lupus nephritis as the primary driver of renal injury.

**Figure 3:**
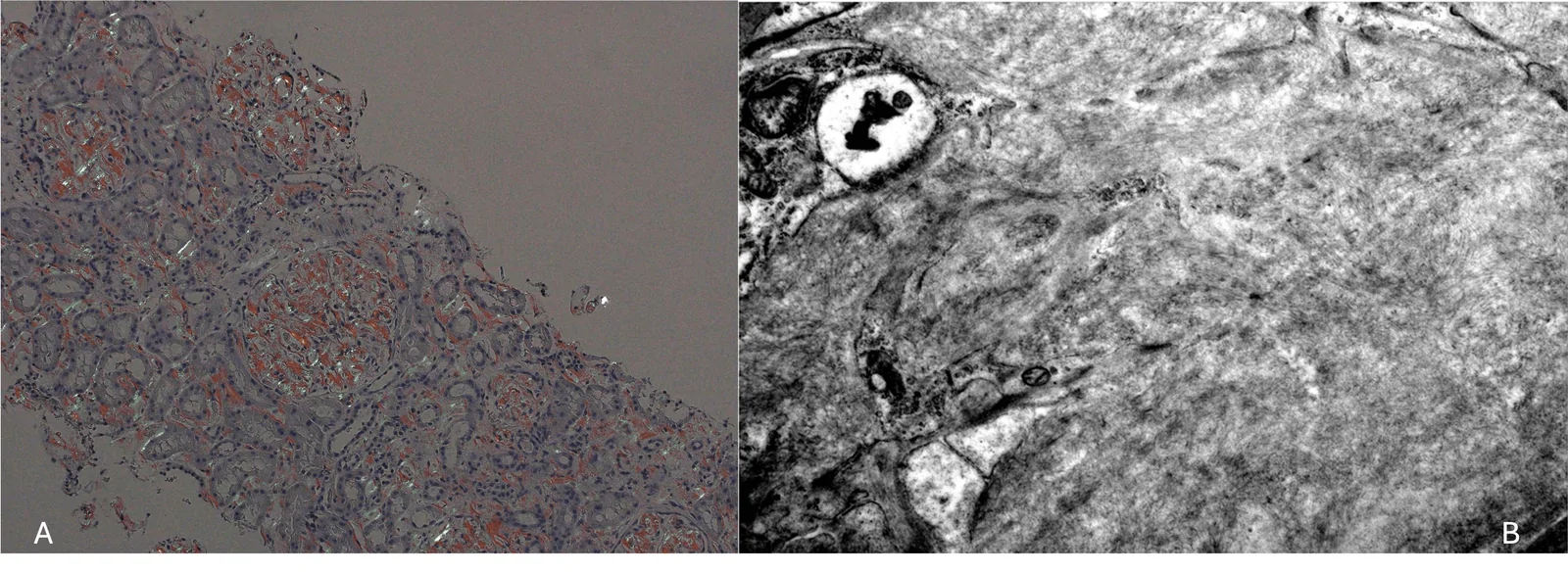
Definitive structural confirmation of amyloid Definitive structural confirmation of amyloid. (A) Glomerular expansion by amorphous, eosinophilic deposits demonstrating characteristic apple-green birefringence under polarized light on Congo red stain. (B) Electron microscopy confirming the presence of numerous, randomly arranged, non-branching amyloid fibrils.

The diagnosis of amyloidosis was definitively confirmed by characteristic apple-green birefringence under polarized light on Congo red stain (Figure 3A) and the presence of numerous amyloid fibrils on electron microscopy (Figure 3B).

A subsequent bone marrow biopsy verified that the systemic amyloidosis was driven by multiple myeloma; however, exact percentages of clonal plasma cells and cytogenetic/flow cytometry data could not be retrieved from the accessed records. In month two, his renal function manifested severe proteinuria (urinary protein: 957.3 mg/dL) and an estimated glomerular filtration rate (eGFR) of 68 mL/min/1.73m².

Therapeutic interventions

The patient’s oncologic management vigorously targeted the aberrant plasma cell clone. In month five, he started induction chemotherapy consisting of carfilzomib, daratumumab, and lenalidomide, a regimen briefly complicated by a marked BNP elevation (3088 pg/mL) and worsening peripheral edema (4+), which required aggressive diuresis.

In month eight, the patient successfully underwent an ASCT. He subsequently received CAR T-cell therapy with cilta-cel in month 14 to attain deep and durable clonal eradication in the setting of his complex disease overlap. A restaging bone marrow biopsy performed in month 16 demonstrated complete marrow remission, prompting the discontinuation of all active chemotherapeutic regimens. He was started on monthly intravenous immunoglobulin (IVIG) supplementation in month 17 to alleviate prolonged hypogammaglobulinemia following CAR T-cell infusion. Due to the retrospective nature of this case report and limitations in medical record access, specific data regarding the exact clinical trigger for this cellular therapy (e.g., measurable residual disease positivity versus planned off-label consolidation), the institutional framework utilized to approve this off-label sequence, and granular grading of any post-infusion cytokine release syndrome (CRS) or immune effector cell-associated neurotoxicity syndrome (ICANS) and its acute management, were unavailable.

Current clinical status (month 22)

The patient demonstrated a stable clinical trajectory at his most recent follow-up in month 22. He noticed subjective improvement in his bilateral lower extremity edema and denied any cardiopulmonary, gastrointestinal, or genitourinary symptoms. His only neurologic complaint was residual neuropathy attributed to chronic sciatica.

A physical examination demonstrated a blood pressure of 142/70 mmHg, heart rate of 64 bpm, and a BMI of 27.67 kg/m². Extremity examination revealed 1-2+ ankle edema bilaterally, with peripheral pulses equal throughout. Examinations across all other major organ systems were normal.

Laboratory and diagnostic findings

Longitudinal laboratory data showed a substantial renal response. From a peak proteinuria of 957.3 mg/dL in month two, his urinary protein excretion dropped to 9.4 mg/dL by month 22. His eGFR has stabilized at 42 mL/min/1.73m² (CKD Stage 3b). He manifests persistent macrocytic anemia and mild thrombocytopenia, consistent with expected delayed hematopoietic recovery following marrow-ablative and CAR-T therapies (Table 1). Serum electrolytes, calcium, erythrocyte sedimentation rate (ESR), and C-reactive protein (CRP) were stable or within normal limits.

**Table 1:** Relevant laboratory results (month 22) RBC - red blood cells; Hgb - hemoglobin; MCV - mean corpuscular volume; fL - femtoliters; Plt - platelets; WBC - white blood cells; Cr - creatinine; BUN - blood urea nitrogen; eGFR - estimated glomerular filtration rate; CKD - chronic kidney disease; UACR - urine microalbumin-to-creatinine ratio; BNP - B-type natriuretic peptide

Analyte	Result	Reference range	Interpretation
Red blood cells (RBC)	2.76 x 10⁶/µL	4.32 - 5.72 x 10⁶/µL	Low
Hemoglobin (Hgb)	9.3 g/dL	13.2 - 16.6 g/dL	Low
Mean corpuscular volume (MCV)	105 fL	80 - 100 fL	Macrocytosis
Platelets (Plt)	104,000/µL	150,000 - 450,000/µL	Mild thrombocytopenia
White blood cells (WBC)	4,400/µL	4,500 - 11,000/µL	Normal
Creatinine (Cr)	1.72 mg/dL	0.74 - 1.35 mg/dL	Elevated
Blood urea nitrogen (BUN)	78 mg/dL	6 - 24 mg/dL	Elevated
Estimated glomerular filtration rate (eGFR)	42 mL/min/1.73m²	> 90 mL/min/1.73m²	CKD stage 3b
Urinary protein	9.4 mg/dL	0 - 14 mg/dL	Significantly improved (from 957.3 mg/dL)
Urine microalbumin-to-creatinine ratio (UACR)	62.6 mg/g	< 30 mg/g	Elevated
B-type natriuretic peptide (BNP)	347 pg/mL	< 100 pg/mL	Elevated
Uric acid	8.2 mg/dL	3.4 - 7.0 mg/dL	Elevated (history of gout)

Ongoing management

The patient's current pharmacologic regimen is adapted to manage his blood pressure, heart failure risk, gout, and viral prophylaxis (Table 2). Due to his history of amyloidosis and an increased BNP (347 pg/mL), a cardiology referral was scheduled for month 22 to assess for cardiac amyloid infiltration. The results of this specific cardiac imaging were pending and unavailable at the time of this publication.

**Table 2:** Current medication regimen (month 22)

Medication	Dosage	Primary indication
Torsemide	20 mg daily	Volume management / edema
Eplerenone	25 mg daily	Heart failure / hypertension
Carvedilol	6.25 mg twice daily with food	Cardioprotection / hypertension
Losartan	50 mg daily	Renoprotection / hypertension
Allopurinol	100 mg daily	Hyperuricemia (gout)
Acyclovir	800 mg twice daily	Viral prophylaxis
Vitamin B complex	1 tablet daily	Nutritional support / neuropathy

Longitudinal laboratory and organ response tracking

Continuous monitoring of the patient's hematologic, renal, and cardiac parameters depicts the physiological impact of the sequential therapeutic interventions (Table 3). The patient demonstrated severe proteinuria (957.3 mg/dL) prior to the initiation of induction chemotherapy in month two. A dramatic, sustained reduction in urinary protein excretion was observed, reaching a nadir of 9.4 mg/dL by month 22 following induction chemotherapy, ASCT, and subsequent cilta-cel infusion. BNP levels showed significant volatility, peaking during induction chemotherapy before stabilizing at a persistently increased baseline post-CAR-T therapy. Hematologic parameters exhibited expected post-treatment cytopenias, with a significant nadir following cellular therapy. Quantitative serum free light chain (FLC) assays were unavailable in the accessed records to independently verify the depth of the clone eradication via standard consensus criteria.

**Table 3:** Longitudinal tracking of hematologic, renal, and cardiac parameters WBC - white blood cells; Hgb - hemoglobin; Plt - platelets; Cr - creatinine; eGFR - estimated glomerular filtration rate; BNP - B-type natriuretic peptide; ASCT - autologous stem cell transplant; CAR-T - chimeric antigen receptor T-cell; NR - not reported

Timeline	Clinical phase	WBC (4.5-11.0 x10³/µL)	Hgb (13.2-16.6 g/dL)	Plt (150-450 x10³/µL)	Cr (0.74-1.35 mg/dL)	eGFR (>90 mL/min/1.73m²)	Urine protein (0-14 mg/dL)	BNP (<100 pg/mL)
Month 2	Pre-treatment baseline	5.5	9.7	210	1.17	68	957.3	NR
Month 5	Induction chemotherapy	4.7	9.6	281	1.29	61	381.8	3088
Month 6	Pre-ASCT evaluation	7.0	9.4	248	1.29	61	261.9	888
Month 10	Post-ASCT follow-up	7.4	9.2	135	1.79	41	NR	NR
Month 11	Pre-CAR-T evaluation	8.1	9.7	132	1.75	42	95.5	452
Month 15	Post-CAR-T (1 month)	3.3	9.4	90	1.65	45	21.8	184
Month 17	Post-CAR-T (3 months)	2.4	8.7	125	1.75	42	41.0	539
Month 19	Post-CAR-T (5 months)	5.1	9.8	117	1.89	38	26.5	229
Month 22	Post-CAR-T (8 months)	4.4	9.3	104	1.72	42	9.4	347

## Discussion

This case gives captivating insights into the modern management of highly complex plasma cell dyscrasias using advanced cellular therapies, emphasizing the intersections of immuno-oncology, nephrology, and rheumatology.

Diagnostic caveats and profound target-organ response in AHL amyloidosis

AHL amyloidosis is an exceptionally rare histopathological subtype characterized by the co-deposition of heavy and light immunoglobulin fragments [2,3]. Recent literature, such as the 2025 report by Gonzalez-Hernandez et al., further emphasizes the diagnostic rarity of AHL amyloidosis and the critical need for advanced therapeutic sequencing to prevent irreversible end-stage renal disease [8]. Importantly, the diagnosis of AHL amyloidosis in this case was heavily suggested by immunofluorescence. A major limitation of this report is the lack of laser microdissection/mass spectrometry (LMD-MS) to definitively characterize the amyloid subtype, meaning the AHL diagnosis remains presumptive.

Despite this limitation, the longitudinal laboratory data distinctly demonstrates the primary goal of amyloidosis management: rapid cessation of precursor protein production to rescue target organs [9]. The eradication of the plasma cell clone in this patient was associated with a substantial renal improvement [10]. From an immense baseline proteinuria of 957.3 mg/dL, his urinary protein excretion decreased, practically resolving to 9.4 mg/dL by month 22. While the patient's baseline eGFR shifted permanently to a range of 38-45 mL/min/1.73m² (likely manifesting irreversible architectural damage from prior amyloid deposition and cumulative nephrotoxicity from chemotherapy), the halt in protein excretion represents that active, ongoing nephrotic damage has been successfully inhibited, changing the trajectory away from ESRD. Emerging data from early-phase trials, such as the NXC-201 study, increasingly support the efficacy of BCMA-directed CAR T-cell therapy in achieving deep hematologic and organ responses in relapsed/refractory AL amyloidosis, reinforcing the rationale for our therapeutic sequence [11].

Feasibility in autoimmune and overlap syndromes

CAR T-cell therapies efficiently target BCMA-expressing malignant plasma cells. However, because of theoretical risks like severe autoimmune flares and uncontrolled CRS, prior oncology trial protocols excluded patients with autoimmune diseases like SLE from pivotal CAR T-cell therapy trials [12]. This patient successfully achieved marrow remission without precipitating an observable lupus flare. Furthermore, recent systematic reviews of CD19-directed CAR T-cell therapy for refractory SLE have demonstrated high remission rates (81%) with CRS that is almost universally mild, supporting the rationale that controlled autoimmune diatheses do not strictly preclude the administration of cellular therapies [6,7,13].

Dynamics of post-CAR-T cytopenias

The hematologic tracking highlights the classic late-stage effects of sequential ASCT and BCMA-directed cellular therapy. The patient's hematologic baseline was reasonably maintained through initial induction. However, the initiation of cilta-cel prompted a marked drop in blood counts, with white blood cells reaching a nadir of 2400/µL three months post-infusion and platelets dropping to 90,000/µL [14]. The persistent mild thrombocytopenia and macrocytic anemia noted eight months post-infusion highlight the prolonged hematopoietic recovery associated with CAR-T therapies [15]. This physiological state is further aggravated by his underlying CKD, which diminishes endogenous erythropoietin production. Moreover, his dependence on monthly IVIG demonstrates the need to manage prolonged, on-target hypogammaglobulinemia to prevent life-threatening opportunistic infections [16].

Cardiac biomarker volatility and surveillance

A primary driver of mortality in AL and AHL amyloidosis is cardiac involvement [17]. The longitudinal trends in BNP offer important insight into the patient's shifting cardiac and hemodynamic status [18]. The alarming spike during early induction chemotherapy (3088 pg/mL in month five) likely revealed treatment-related fluid shifts and acute volume overload. The BNP trended downward significantly following aggressive diuresis, ASCT, and cellular therapy, but remained persistently elevated above normal limits (ranging between 184 and 539 pg/mL post-CAR-T). This persistent elevation, viewed alongside his history of systemic amyloidosis, validates the absolute need for meticulous, ongoing cardiovascular imaging to rule out slow progression of amyloid infiltrative cardiomyopathy.

## Conclusions

The sequential application of autologous stem cell transplantation and ciltacabtagene autoleucel was associated with continued hematologic and renal improvement in a patient with concurrent multiple myeloma, presumptive immunoglobulin light chain (AL) and heavy-and-light-chain (AHL) amyloidosis, and systemic lupus erythematosus. While encouraging, this is a single case constrained by retrospective data limitations; larger studies or registry data would be needed before changing clinical guidelines to routinely permit the use of advanced immunotherapies in patients with underlying autoimmune diatheses. This report suggests the feasibility of integrating cellular therapies in highly complex overlap syndromes, though definitive causality regarding target-organ reversal cannot be isolated from prior induction and transplant effects. Despite these notable clinical outcomes, this aggressive therapeutic sequence demands meticulous, multidisciplinary vigilance. Prolonged post-CAR-T cytopenias and on-target hypogammaglobulinemia necessitate meticulous supportive care, including routine intravenous immunoglobulin supplementation, while persistent cardiac biomarker elevation mandates continuous cardiovascular surveillance to rule out infiltrative cardiomyopathy. As cellular therapies expand into broader populations, further prospective studies and multi-institutional registries are urgently required to optimize toxicity management and establish standardized survivorship guidelines for patients with overlapping plasma cell dyscrasias and autoimmune diseases.

## References

[1] Merlini G, Dispenzieri A, Sanchorawala V, Schönland SO, Palladini G, Hawkins PN, Gertz MA (2018). Systemic immunoglobulin light chain amyloidosis. Nat Rev Dis Primers.

[2] Picken MM (2020). The pathology of amyloidosis in classification: a review. Acta Haematol.

[3] Nasr SH, Said SM, Valeri AM (2013). The diagnosis and characteristics of renal heavy-chain and heavy/light-chain amyloidosis and their comparison with renal light-chain amyloidosis. Kidney Int.

[4] Anders HJ, Saxena R, Zhao MH (2020). Lupus nephritis. Nat Rev Dis Primers.

[5] Palladini G, Merlini G (2016). What is new in diagnosis and management of light chain amyloidosis?. Blood.

[6] Martin T, Usmani SZ, Berdeja JG (2023). Ciltacabtagene autoleucel, an anti-B-cell maturation antigen chimeric antigen receptor T-cell therapy, for relapsed/refractory multiple myeloma: CARTITUDE-1 2-year follow-up. J Clin Oncol.

[7] Berdeja JG, Madduri D, Usmani SZ (2021). Ciltacabtagene autoleucel, a B-cell maturation antigen-directed chimeric antigen receptor T-cell therapy in patients with relapsed or refractory multiple myeloma (CARTITUDE- 1): a phase 1b/2 open-label study. Lancet.

[8] Gonzalez-Hernandez DR, Hanouneh M, Cervantes CE (2025). An atypical cause of amyloidosis: a case of combined heavy and light chain amyloidosis. BMC Nephrol.

[9] Gertz MA, Comenzo R, Falk RH (2005). Definition of organ involvement and treatment response in immunoglobulin light chain amyloidosis (AL): a consensus opinion from the 10th International Symposium on Amyloid and Amyloidosis. Am J Hematol.

[10] Palladini G, Hegenbart U, Milani P (2014). A staging system for renal outcome and early markers of renal response to chemotherapy in AL amyloidosis. Blood.

[11] Landau HJ, Raza S, Rosenberg A (2026). First 23-patient safety and efficacy data from Nexicart-2, the first US trial of CAR-T in R/R light chain (AL) amyloidosis, NXC-201. Transplant Cell Ther.

[12] Jaggers JL, Giri S, Klepin HD (2021). Characterizing inclusion and exclusion criteria in clinical trials for chimeric antigen receptor (CAR) T-cell therapy among adults with hematologic malignancies. J Geriatr Oncol.

[13] Sayed OA, Ellebedy M, Abu-Alsaud MA, Meligy AI, Gheita TA (2026). CAR T cell therapy efficacy and safety in SLE: a systematic review and pooled analysis of 47 patients across 10 studies. Naunyn Schmiedebergs Arch Pharmacol.

[14] Jain T, Knezevic A, Pennisi M (2020). Hematopoietic recovery in patients receiving chimeric antigen receptor T-cell therapy for hematologic malignancies. Blood Adv.

[15] Dima D, Logue JM, Waqar SH (2026). Cytopenias and infections following ciltacabtagene autoleucel in heavily pretreated relapsed or refractory multiple myeloma. Haematologica.

[16] Wudhikarn K, Perales MA (2022). Infectious complications, immune reconstitution, and infection prophylaxis after CD19 chimeric antigen receptor T-cell therapy. Bone Marrow Transplant.

[17] Grogan M, Dispenzieri A, Gertz MA (2017). Light-chain cardiac amyloidosis: strategies to promote early diagnosis and cardiac response. Heart.

[18] Palladini G, Dispenzieri A, Gertz MA (2012). New criteria for response to treatment in immunoglobulin light chain amyloidosis based on free light chain measurement and cardiac biomarkers: impact on survival outcomes. J Clin Oncol.

